# Supplementary material for: Changes in the Proteomic Profile After Audiogenic Kindling in the Inferior Colliculus of the GASH/Sal Model of Epilepsy
Source: Int J Mol Sci. 2025 Mar 5;26(5):2331. doi: 10.3390/ijms26052331 (PMC11900993; doi:10.3390/ijms26052331)
Supplement: Supplementary file 1 [file ijms-26-02331-s001.zip › Supplementary Figures and Tables Captions.pdf]

Supplementary Figure S1:

Metabolic epileptic disorders pathway. 14 significant down-regulated pathways, grouped by theme, are displayed at the top of the image (3 of them do not belong to any cluster). Title of the theme corresponds to the most significant term. Below, the metabolic pathway related with epilepsy disorders is illustrated. Green hits correspond with the lowest values of LogFoldChange, while red ones correspond with highest values. Grey boxes correspond with no hits.

Supplementary Figure S2:

PPI (Protein-Protein Interaction) network. Significant proteins from Module green were clustered in 11 networks. The employed layout distribution for the figure was "yFiles Circular layout" from Cytoscape. The names of the proteins and their descriptions can be found in the Supplementary table 7.

Supplementary Figure S3:

Cluster 1 network representing epilepsy-combined scores from Disgenet and Disease 2.0 databases. Highest values are displayed in red while lowest ones correspond to blue color. The employed layout distribution for the figure was "yFiles Circular layout" from Cytoscape.

Supplementary Table S1:

Dunn's multiple comparison study among all hamsters submitted to the sAUK protocol. The results indicate the existence of two GASH/Sal subgroups clearly different from each other (adjusted $p < 0.05$).

Supplementary Table S2:

Comparison of the mean severity index between responders sAUK (GASH.sAUK.R) and non-responders sAUK hamsters (GASH.sAUK.NR) for each stimulus (1-45). A Mann-Whitney test following the Benjamini, Krieger and Yekutieli method was performed. Data was considered significant when $p < 0.05$ (*).

Supplementary Table S3:

Results of cytokines investigated in the plasma of GASH naïve compared to GASH sAUK.NR hamsters. All significant proteins were detected at higher levels in GASH/Sal non responder animals except for IGF-1 alpha, which was at lower levels in the stimulated group. Abbreviations: GCSF (Granulocyte Colony-Stimulating Factor), IFN-gamma (Interferon-gamma), IGF-1 (Insulin-like Growth Factor 1), IL-1 alpha (Interleukin-1 alpha), IL-1 beta (Interleukin-1 beta), IL-4 (Interleukin-4), IL-6 (Interleukin-6), IL-10 (Interleukin-10), KC (Keratinocyte Chemoattractant), LIX (LPS-induced CXC chemokine), MCP-1 (Monocyte Chemoattractant Protein 1), M-CSF (Macrophage Colony-stimulating Factor), MIP-1 alpha (Macrophage Inflammatory Protein 1 alpha), RAGE (Receptor for Advanced Glycation End Products), SDF-1 (Stromal Cell-Derived Factor 1), TARC (Thymus and Activation-Regulated Chemokine), TGF-beta (Transforming Growth Factor beta), TNF-alpha (Tumor Necrosis Factor alpha), VEGF-A (Vascular Endothelial Growth Factor A)

Supplementary Table S4:

Univariate t-test analysis result for the comparison between GASH.sAuk.NR and GASH.naïve groups. (Sheet 1) Statistics for all Mesocricetus auratus proteins (4085) with human homologous genes (Column 2). Isoforms were excluded. (Sheet (2) 159 Differentially expressed proteins (DEPs) (p-value < 0.05). Column 2 represents Mesocricetus auratus protein identifiers while column 3 represents human homologous genes.

Supplementary Table S5:

Overall result of GSEA analysis for the comparison between GASH.sAuk.NR and GASH.naïve groups. (Sheet 1) No significant up-regulated pathways were found. (Sheet 2) 14 significant down-regulated pathways were found (FDR ≤ 0.05). Column names abbreviations: ES (Enrichment Score), NES (Normalized Enrichment Score), NOM (Nominal) p-value, FDR (False Discovery Rate) q-value.

Supplementary Table S6:

WGCNA result for the comparison between GASH.sAuk.NR and GASH.naïve groups. The table presents the protein significance associated with the variable 'epilepsy status' (p-value) and the probability of protein Module Membership (MM).

Supplementary Table S7:

Protein-Protein Interaction clusters for significant proteins in Module green. 11 clusters were found at a high cutoff (0.7) for the interaction confidence score.

Supplementary Table S8:

Cluster 1 epilepsy-related scores. The last column of the table presents the average score for each protein, calculated from the Disgenet and Disease 2.0 databases.
