# Supplementary material for: Changes in the Proteomic Profile After Audiogenic Kindling in the Inferior Colliculus of the GASH/Sal Model of Epilepsy
Source: Int J Mol Sci. 2025 Mar 5;26(5):2331. doi: 10.3390/ijms26052331 (PMC11900993; doi:10.3390/ijms26052331)
Supplement: Supplementary file 1 [file ijms-26-02331-s001.zip › Table S1.pdf]

Dunn's multiple comparisons test	Mean rank difference	Significant?	Summary	Adjusted *p*-value
4618 vs. 4623	95.83	No	ns	>0.9999
4618 vs. 4626	211.6	Yes	**	0.0057
4618 vs. 4625	281.9	Yes	****	<0.0001
4618 vs. 4631	151.3	No	ns	0.542
4618 vs. 4627	-19	No	ns	>0.9999
4618 vs. 5017	199.7	Yes	*	0.0155
4618 vs. 5020	226.1	Yes	**	0.0016
4618 vs. 5022	177.6	No	ns	0.0874
4618 vs. 5019	22.41	No	ns	>0.9999
4618 vs. 5026	237.3	Yes	***	0.0005
4618 vs. 5027	77.43	No	ns	>0.9999
4618 vs. 5030	350.7	Yes	****	<0.0001
4618 vs. 5031	64.78	No	ns	>0.9999
4618 vs. 5038	304.2	Yes	****	<0.0001
4618 vs. 5040	251.3	Yes	***	0.0001
4618 vs. 5043	293	Yes	****	<0.0001
4618 vs. 5054	329.8	Yes	****	<0.0001
4618 vs. 5058	193.9	Yes	*	0.025
4618 vs. 5060	232.2	Yes	***	0.0009
4623 vs. 4626	115.7	No	ns	>0.9999
4623 vs. 4625	186	Yes	*	0.0463
4623 vs. 4631	55.43	No	ns	>0.9999
4623 vs. 4627	-114.8	No	ns	>0.9999
4623 vs. 5017	103.9	No	ns	>0.9999
4623 vs. 5020	130.3	No	ns	>0.9999
4623 vs. 5022	81.79	No	ns	>0.9999
4623 vs. 5019	-73.42	No	ns	>0.9999
4623 vs. 5026	141.4	No	ns	>0.9999
4623 vs. 5027	-18.4	No	ns	>0.9999
4623 vs. 5030	254.9	Yes	****	<0.0001
4623 vs. 5031	-31.06	No	ns	>0.9999
4623 vs. 5038	208.3	Yes	**	0.0076
4623 vs. 5040	155.5	No	ns	0.4118
4623 vs. 5043	197.2	Yes	*	0.0191
4623 vs. 5054	234	Yes	***	0.0007
4623 vs. 5058	98.06	No	ns	>0.9999
4623 vs. 5060	136.3	No	ns	>0.9999
4626 vs. 4625	70.31	No	ns	>0.9999
4626 vs. 4631	-60.29	No	ns	>0.9999
4626 vs. 4627	-230.6	Yes	**	0.001
4626 vs. 5017	-11.81	No	ns	>0.9999
4626 vs. 5020	14.54	No	ns	>0.9999

4626 vs. 5022	-33.93	No	ns	>0.9999
4626 vs. 5019	-189.1	Yes	*	0.0363
4626 vs. 5026	25.72	No	ns	>0.9999
4626 vs. 5027	-134.1	No	ns	>0.9999
4626 vs. 5030	139.2	No	ns	>0.9999
4626 vs. 5031	-146.8	No	ns	0.7211
4626 vs. 5038	92.61	No	ns	>0.9999
4626 vs. 5040	39.76	No	ns	>0.9999
4626 vs. 5043	81.47	No	ns	>0.9999
4626 vs. 5054	118.2	No	ns	>0.9999
4626 vs. 5058	-17.67	No	ns	>0.9999
4626 vs. 5060	20.62	No	ns	>0.9999
4625 vs. 4631	-130.6	No	ns	>0.9999
4625 vs. 4627	-300.9	Yes	****	<0.0001
4625 vs. 5017	-82.12	No	ns	>0.9999
4625 vs. 5020	-55.77	No	ns	>0.9999
4625 vs. 5022	-104.2	No	ns	>0.9999
4625 vs. 5019	-259.5	Yes	****	<0.0001
4625 vs. 5026	-44.59	No	ns	>0.9999
4625 vs. 5027	-204.4	Yes	*	0.0105
4625 vs. 5030	68.88	No	ns	>0.9999
4625 vs. 5031	-217.1	Yes	**	0.0035
4625 vs. 5038	22.3	No	ns	>0.9999
4625 vs. 5040	-30.56	No	ns	>0.9999
4625 vs. 5043	11.16	No	ns	>0.9999
4625 vs. 5054	47.93	No	ns	>0.9999
4625 vs. 5058	-87.98	No	ns	>0.9999
4625 vs. 5060	-49.69	No	ns	>0.9999
4631 vs. 4627	-170.3	No	ns	0.1492
4631 vs. 5017	48.48	No	ns	>0.9999
4631 vs. 5020	74.83	No	ns	>0.9999
4631 vs. 5022	26.36	No	ns	>0.9999
4631 vs. 5019	-128.9	No	ns	>0.9999
4631 vs. 5026	86.01	No	ns	>0.9999
4631 vs. 5027	-73.83	No	ns	>0.9999
4631 vs. 5030	199.5	Yes	*	0.0159
4631 vs. 5031	-86.49	No	ns	>0.9999
4631 vs. 5038	152.9	No	ns	0.4876
4631 vs. 5040	100	No	ns	>0.9999
4631 vs. 5043	141.8	No	ns	0.9842
4631 vs. 5054	178.5	No	ns	0.0817
4631 vs. 5058	42.62	No	ns	>0.9999
4631 vs. 5060	80.91	No	ns	>0.9999
4627 vs. 5017	218.7	Yes	**	0.003
4627 vs. 5020	245.1	Yes	***	0.0003

4627 vs. 5022	196.6	Yes	*	0.02
4627 vs. 5019	41.41	No	ns	>0.9999
4627 vs. 5026	256.3	Yes	****	<0.0001
4627 vs. 5027	96.43	No	ns	>0.9999
4627 vs. 5030	369.7	Yes	****	<0.0001
4627 vs. 5031	83.78	No	ns	>0.9999
4627 vs. 5038	323.2	Yes	****	<0.0001
4627 vs. 5040	270.3	Yes	****	<0.0001
4627 vs. 5043	312	Yes	****	<0.0001
4627 vs. 5054	348.8	Yes	****	<0.0001
4627 vs. 5058	212.9	Yes	**	0.0051
4627 vs. 5060	251.2	Yes	***	0.0001
5017 vs. 5020	26.36	No	ns	>0.9999
5017 vs. 5022	-22.12	No	ns	>0.9999
5017 vs. 5019	-177.3	No	ns	0.0893
5017 vs. 5026	37.53	No	ns	>0.9999
5017 vs. 5027	-122.3	No	ns	>0.9999
5017 vs. 5030	151	No	ns	0.5513
5017 vs. 5031	-135	No	ns	>0.9999
5017 vs. 5038	104.4	No	ns	>0.9999
5017 vs. 5040	51.57	No	ns	>0.9999
5017 vs. 5043	93.28	No	ns	>0.9999
5017 vs. 5054	130.1	No	ns	>0.9999
5017 vs. 5058	-5.856	No	ns	>0.9999
5017 vs. 5060	32.43	No	ns	>0.9999
5020 vs. 5022	-48.48	No	ns	>0.9999
5020 vs. 5019	-203.7	Yes	*	0.0112
5020 vs. 5026	11.18	No	ns	>0.9999
5020 vs. 5027	-148.7	No	ns	0.64
5020 vs. 5030	124.6	No	ns	>0.9999
5020 vs. 5031	-161.3	No	ns	0.2784
5020 vs. 5038	78.07	No	ns	>0.9999
5020 vs. 5040	25.21	No	ns	>0.9999
5020 vs. 5043	66.92	No	ns	>0.9999
5020 vs. 5054	103.7	No	ns	>0.9999
5020 vs. 5058	-32.21	No	ns	>0.9999
5020 vs. 5060	6.078	No	ns	>0.9999
5022 vs. 5019	-155.2	No	ns	0.4191
5022 vs. 5026	59.66	No	ns	>0.9999
5022 vs. 5027	-100.2	No	ns	>0.9999
5022 vs. 5030	173.1	No	ns	0.1215
5022 vs. 5031	-112.8	No	ns	>0.9999
5022 vs. 5038	126.5	No	ns	>0.9999
5022 vs. 5040	73.69	No	ns	>0.9999
5022 vs. 5043	115.4	No	ns	>0.9999

5022 vs. 5054	152.2	No	ns	0.511
5022 vs. 5058	16.27	No	ns	>0.9999
5022 vs. 5060	54.56	No	ns	>0.9999
5019 vs. 5026	214.9	Yes	**	0.0043
5019 vs. 5027	55.02	No	ns	>0.9999
5019 vs. 5030	328.3	Yes	****	<0.0001
5019 vs. 5031	42.37	No	ns	>0.9999
5019 vs. 5038	281.8	Yes	****	<0.0001
5019 vs. 5040	228.9	Yes	**	0.0012
5019 vs. 5043	270.6	Yes	****	<0.0001
5019 vs. 5054	307.4	Yes	****	<0.0001
5019 vs. 5058	171.5	No	ns	0.1368
5019 vs. 5060	209.8	Yes	**	0.0067
5026 vs. 5027	-159.8	No	ns	0.3077
5026 vs. 5030	113.5	No	ns	>0.9999
5026 vs. 5031	-172.5	No	ns	0.1271
5026 vs. 5038	66.89	No	ns	>0.9999
5026 vs. 5040	14.03	No	ns	>0.9999
5026 vs. 5043	55.74	No	ns	>0.9999
5026 vs. 5054	92.52	No	ns	>0.9999
5026 vs. 5058	-43.39	No	ns	>0.9999
5026 vs. 5060	-5.1	No	ns	>0.9999
5027 vs. 5030	273.3	Yes	****	<0.0001
5027 vs. 5031	-12.66	No	ns	>0.9999
5027 vs. 5038	226.7	Yes	**	0.0015
5027 vs. 5040	173.9	No	ns	0.115
5027 vs. 5043	215.6	Yes	**	0.004
5027 vs. 5054	252.4	Yes	***	0.0001
5027 vs. 5058	116.5	No	ns	>0.9999
5027 vs. 5060	154.7	No	ns	0.4322
5030 vs. 5031	-286	Yes	****	<0.0001
5030 vs. 5038	-46.58	No	ns	>0.9999
5030 vs. 5040	-99.43	No	ns	>0.9999
5030 vs. 5043	-57.72	No	ns	>0.9999
5030 vs. 5054	-20.94	No	ns	>0.9999
5030 vs. 5058	-156.9	No	ns	0.3759
5030 vs. 5060	-118.6	No	ns	>0.9999
5031 vs. 5038	239.4	Yes	***	0.0004
5031 vs. 5040	186.5	Yes	*	0.0445
5031 vs. 5043	228.2	Yes	**	0.0013
5031 vs. 5054	265	Yes	****	<0.0001
5031 vs. 5058	129.1	No	ns	>0.9999
5031 vs. 5060	167.4	No	ns	0.1828
5038 vs. 5040	-52.86	No	ns	>0.9999
5038 vs. 5043	-11.14	No	ns	>0.9999

5038 vs. 5054	25.63	No	ns	>0.9999
5038 vs. 5058	-110.3	No	ns	>0.9999
5038 vs. 5060	-71.99	No	ns	>0.9999
5040 vs. 5043	41.71	No	ns	>0.9999
5040 vs. 5054	78.49	No	ns	>0.9999
5040 vs. 5058	-57.42	No	ns	>0.9999
5040 vs. 5060	-19.13	No	ns	>0.9999
5043 vs. 5054	36.78	No	ns	>0.9999
5043 vs. 5058	-99.13	No	ns	>0.9999
5043 vs. 5060	-60.84	No	ns	>0.9999
5054 vs. 5058	-135.9	No	ns	>0.9999
5054 vs. 5060	-97.62	No	ns	>0.9999
5058 vs. 5060	38.29	No	ns	>0.9999

Table S1. Dunn's multiple comparison study among all hamsters submitted to the sAUK protocol. The results indicate the existence of two GASH/Sal subgroups clearly different from each other (adjusted $p < 0.05$). * $p < 0.05$; ** $p < 0.01$; *** $p < 0.001$, **** $p < 0.0001$; n.s, non-significant.
