# Supplementary material for: Changes in the Proteomic Profile After Audiogenic Kindling in the Inferior Colliculus of the GASH/Sal Model of Epilepsy
Source: Int J Mol Sci. 2025 Mar 5;26(5):2331. doi: 10.3390/ijms26052331 (PMC11900993; doi:10.3390/ijms26052331)
Supplement: Supplementary file 1 [file ijms-26-02331-s001.zip › Table S2.pdf]

Stimuli	Discovery	*p*-value	Mean rank of R- sAUK	Mean rank of NR- sAUK	Mean rank diff.	Mann-Whitney U	*q*-value
1	No	>0.99999	10.5	10.5	0	42	0.42
2	No	>0.99999	10.5	10.5	0	42	0.42
3	No	>0.99999	10.5	10.5	0	42	0.42
4	No	>0.99999	10.5	10.5	0	42	0.42
5	No	>0.99999	10.5	10.5	0	42	0.42
6	No	>0.99999	10.5	10.5	0	42	0.42
7	No	>0.99999	11	10.29	0.7143	39	0.42
8	No	>0.99999	10.5	10.5	0	42	0.42
9	No	>0.99999	11	10.29	0.7143	39	0.42
10	No	>0.99999	10.5	10.5	0	42	0.42
11	No	0.213493	13	9.429	3.571	27	0.122273
12	No	0.260449	13	9.429	3.571	27	0.144446
13	No	0.201754	13	9.429	3.571	27	0.119161
14	No	0.267492	12.5	9.643	2.857	30	0.144446
15	**Yes**	**0.051496**	**14**	**9**	**5**	**21**	**0.033561**
16	Yes	0.018318	15	8.571	6.429	15	0.015737
17	No	0.114886	13.5	9.214	4.286	24	0.072378
18	Yes	0.027348	14.58	8.75	5.833	17.5	0.019143
19	Yes	0.01824	15	8.571	6.429	15	0.015737
20	Yes	0.004257	16	8.143	7.857	9	0.008707
21	Yes	0.019608	15	8.571	6.429	15	0.016113
22	Yes	0.004696	15.75	8.25	7.5	10.5	0.008707
23	Yes	0.033153	14.83	8.643	6.19	16	0.022378
24	Yes	0.024561	15	8.571	6.429	15	0.018568
25	Yes	0.000181	17	7.714	9.286	3	0.001707
26	Yes	0.001058	16.5	7.929	8.571	6	0.002856
27	Yes	0.000335	17	7.714	9.286	3	0.00195
28	Yes	0.013313	15.42	8.393	7.024	12.5	0.013243
29	Yes	0.00983	15.58	8.321	7.262	11.5	0.010928
30	Yes	0.005315	15.92	8.179	7.738	9.5	0.008707
31	Yes	0.004773	16	8.143	7.857	9	0.008707
32	Yes	0.000129	17.17	7.643	9.524	2	0.001707
33	Yes	0.017389	15.33	8.429	6.905	13	0.015737
34	Yes	0.009159	15.5	8.357	7.143	12	0.010819
35	Yes	0.000516	16.75	7.821	8.929	4.5	0.00195
36	Yes	0.00645	15.33	8.429	6.905	13	0.008707
37	Yes	0.006218	15.17	8.5	6.667	14	0.008707
38	No	0.137539	13.5	9.214	4.286	24	0.083854
39	Yes	0.000516	16.5	7.929	8.571	6	0.00195
40	Yes	0.005676	15.33	8.429	6.905	13	0.008707
41	Yes	0.013106	14.92	8.607	6.31	15.5	0.013243
42	Yes	0.000903	16.5	7.929	8.571	6	0.002844
43	Yes	0.025671	14.33	8.857	5.476	19	0.018661
44	Yes	0.006914	15.08	8.536	6.548	14.5	0.008712
45	Yes	0.02402	15	8.571	6.429	15	0.018568

Table S2. Comparison of the mean severity index between responders sAUK (GASH.sAUK.R) and non-responders sAUK hamsters (GASH.sAUK.NR) for each stimulus (1-45). A Mann-Whitney test following the Benjamini, Krieger and Yekutieli method was performed. Data was considered significant when *p*-value < 0.05.
