# Supplementary material for: Changes in the Proteomic Profile After Audiogenic Kindling in the Inferior Colliculus of the GASH/Sal Model of Epilepsy
Source: Int J Mol Sci. 2025 Mar 5;26(5):2331. doi: 10.3390/ijms26052331 (PMC11900993; doi:10.3390/ijms26052331)
Supplement: Supplementary file 1 [file ijms-26-02331-s001.zip › Table S3.pdf]

GASH naïve vs. GASH sAUK NR		
**Cytokine**	**GASH.sAUK.NR**	***p*-value**
Fas ligand	2.353 ± 0.481	*N. S*
Fracktalkina	1.793 ± 0.09406	$p < 0.01$
GCSF	3.859 ± 0.7214	$p < 0.01$
IFN- gamma	2.944 ± 0.4409	$p < 0.01$
IGF-1	0.3447 ± 0.09273	$p < 0.01$
IL-10	3.029 ± 0.4959	$p < 0.01$
IL-1 alpha	2.789 ± 0.1314	$p < 0.01$
IL-1 beta	3.073 ± 0.4255	$p < 0.01$
IL-4	0.3941 ± 0.05795	*N. S*
IL-6	2.072 ± 0.2957	$p < 0.01$
KC	3.417 ± 0.2613	$p < 0.01$
LIX	1.243 ± 0.08047	*N. S*
MCP-1	1.582 ± 0.0957	$p < 0.05$
M-CSF	1.706 ± 0.1257	$p < 0.05$
MIP-1 alpha	2.079 ± 0.1307	$p < 0.001$
RAGE	2.278 ± 0.1582	$p < 0.01$
TARC	2.189 ± 0.1534	$p < 0.01$
SDF-1 alpha	0.7023 ± 0.1952	*N. S*
TGF-beta	2.82 ± 0.3576	$p < 0.001$
TNF-alpha	2.495 ± 0.4296	$p < 0.01$
VEGF-A	7. 633 ± 1.289	$p < 0.001$

Table S3. Results of cytokines investigated in the plasma of GASH naïve compared to GASH sAUK.NR hamsters. All significant proteins were detected at higher levels in GASH/Sal non responder animals except for IGF-1 alpha, which was at lower levels in the stimulated group. N.S, non-significant. Cyokine abbreviations: GCSF: Granulocyte colony-stimulating factor; IFN-gamma: Interferon gamma; IGF-1: Insulin-like growth factor 1; IL-10: Interleukin 10; IL-1 alpha: Interleukin 1 alpha; IL-1beta: Interleukin 1 beta; IL-4: Interleukin 4; IL-6: Interleukin 6; KC: Keratinocyte chemoattractant; LIX: LPS-induced CXC chemokine; MCP-1: Monocyte Chemoattractant Protein 1; M-CSF: Macrophage Colony-stimulating Factor; MIP-1alpha: Macrophage Inflammatory Protein 1 alpha; RAGE: Receptor for Advanced Glycation End products; TARC: Thymus and Activation-Regulated Chemokine; SDF-1 alpha: Stromal Cell-Derived Factor 1 alpha; TGF-beta: Transforming growth factor beta; TNF-alpha: Tumor Necrosis Factor alpha; VEGF-A: Vascular Endothelial Growth Factor A.
