# Supplementary figures and images for: Changes in the Proteomic Profile After Audiogenic Kindling in the Inferior Colliculus of the GASH/Sal Model of Epilepsy
Source: Int J Mol Sci. 2025 Mar 5;26(5):2331. doi: 10.3390/ijms26052331 (PMC11900993; doi:10.3390/ijms26052331)

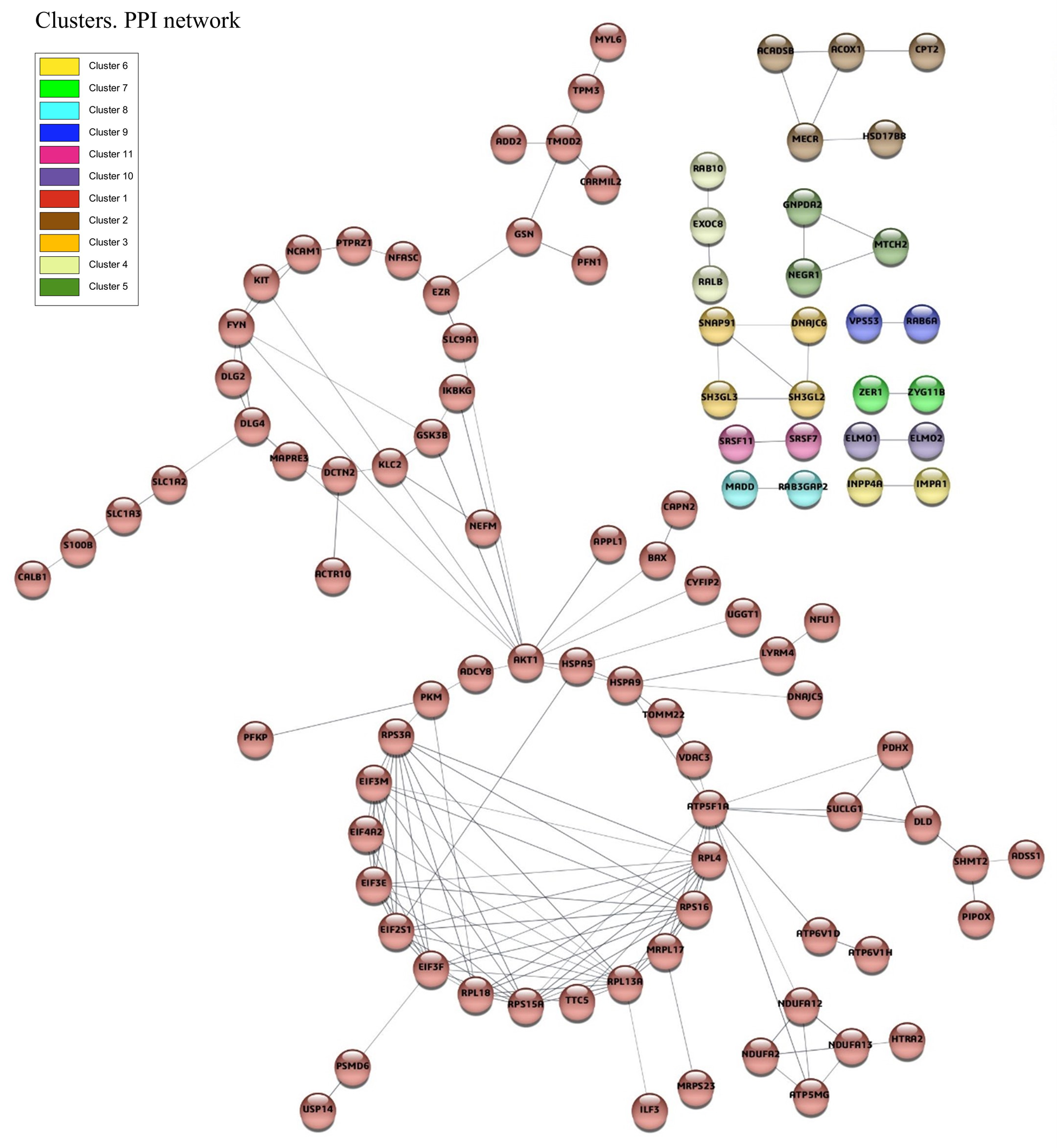

Supplement: Supplementary file 1 [file ijms-26-02331-s001.zip › Figure S2.tiff]

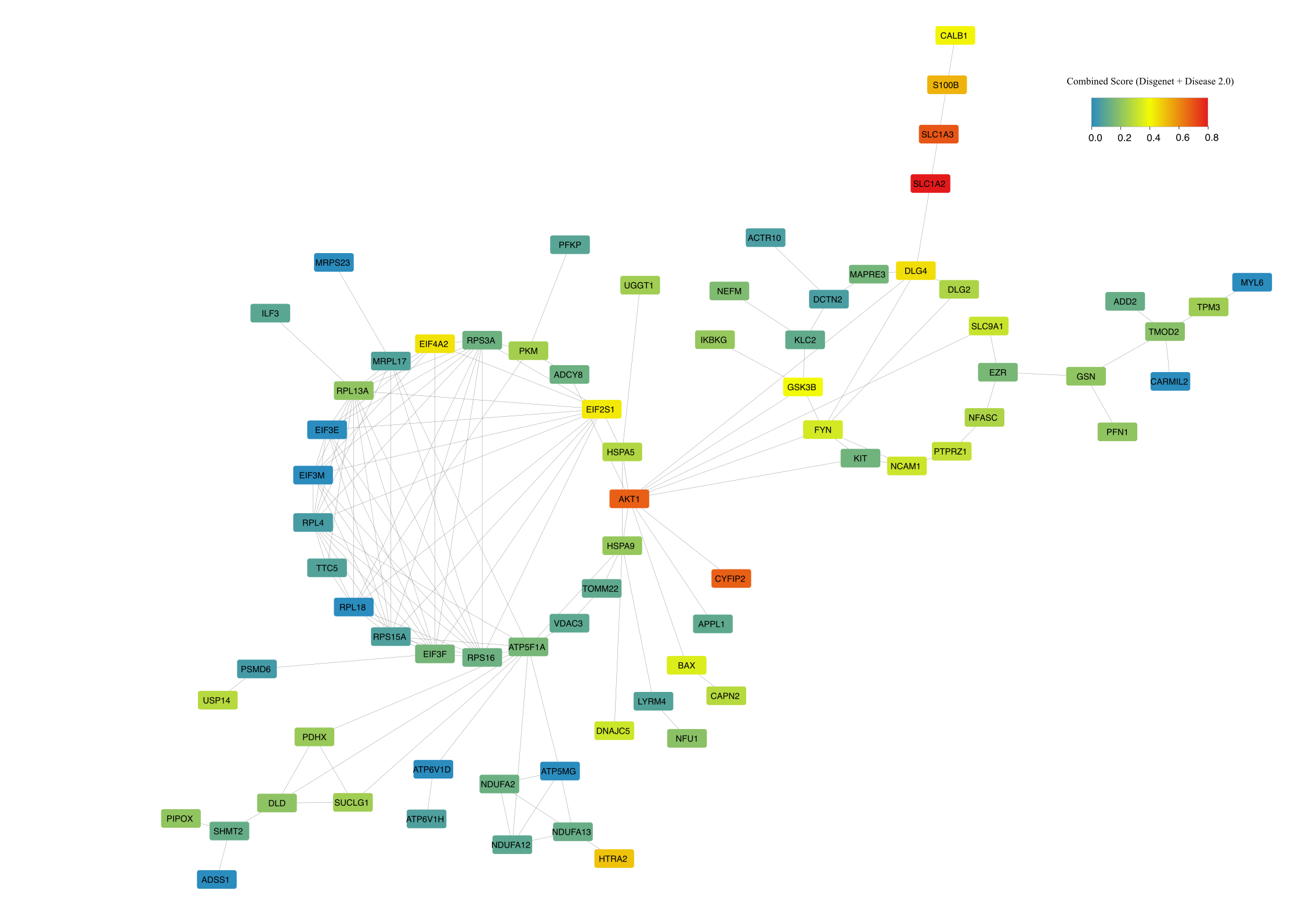

Supplement: Supplementary file 1 [file ijms-26-02331-s001.zip › Figure S3.tiff]
